# Inhibition of the calcium-sensing receptor by extracellular phosphate ions and by intracellular phosphorylation

**DOI:** 10.3389/fphys.2023.1154374

**Published:** 2023-03-31

**Authors:** Patricia P. Centeno, Lenah S. Binmahfouz, Khaleda Alghamdi, Donald T. Ward

**Affiliations:** ^1^ Faculty of Biology, Medicine and Health, The University of Manchester, Manchester, United Kingdom; ^2^ Department of Pharmacology and Toxicology, Faculty of Pharmacy, King Abdulaziz University, Jeddah, Saudi Arabia

**Keywords:** calcium-sensing receptor, parathyroid hormone, phosphate-sensing, secondary hyperparathyroidism, receptor phosphorylation

## Abstract

As both a sensor of extracellular calcium (Ca^2+^_o_) concentration and a key controller of Ca^2+^_o_ homeostasis, one of the most interesting properties of the calcium-sensing receptor (CaR) is its sensitivity to, and modulation by, ions and small ligands other than Ca^2+^. There is emerging evidence that extracellular phosphate can act as a partial, non-competitive CaR antagonist to modulate parathyroid hormone (PTH) secretion, thus permitting the CaR to integrate mineral homeostasis more broadly. Interestingly, phosphorylation of certain intracellular CaR residues can also inhibit CaR responsiveness. Thus, negatively charged phosphate can decrease CaR activity both extracellularly (*via* association with arginine) and intracellularly (*via* covalent phosphorylation).

## Introduction

### Calcium and phosphate homeostasis

Ca^2+^ is the fifth most abundant element both in the human body and the Earth’s crust. Of the environmentally available ions, Ca^2+^ is the most reactive and thus has been selected as a signalling molecule that can control millisecond duration intracellular events, for example, heartbeat, memory and neuro- and hormone secretion and locomotion (Williams, 2006). Outside of the cell, Ca^2+^ serves more of a structural role, both in tight junctions but most notably as a core component of bone hydroxyapatite. As a result of these differential functions, the 10^4^ M extracellular *versus* intracellular Ca^2+^ (Ca^2+^_i_) concentration gradient must be restored after each intracellular Ca^2+^ (Ca^2+^_i_) signal has had its effect. Otherwise, sustained rises in Ca^2+^_i_ concentration will drive Ca^2+^ overload and cell death (Duchen, 2000).

The other major component of hydroxyapatite is inorganic phosphate (PO_4_). Phosphorous is the sixth most abundant element in the body, though is much rarer in the terrestrial environment than calcium. PO_4_ is the most abundant intracellular anion and exists in millimolar concentrations inside the cell both as a free ion (involved in pH buffering) and as a critical component of the energy-releasing molecules phosphocreatine and adenosine triphosphate (ATP) as well as the other nucleotides. The protein kinase-mediated addition of negatively-charged PO_4_ groups to the sidechains of certain tyrosine and serine/threonine residues can effect local conformational changes in protein tertiary structure that can produce profound functional changes in those proteins (Hunter, 2012). Indeed, protein phosphorylation and Ca^2+^_i_ mobilisation together represent two of the most fundamental mechanisms of intracellular signalling in nature. Therefore, any protein that plays a key role in controlling the total availability of both Ca^2+^ and PO_4_ in the body is of critical importance for both extracellular structure and intracellular signalling.

The CaR is a homodimeric G protein-coupled receptor (GPCR; family C) expressed with greatest abundance in organs involved in mineral ion homeostasis, specifically the parathyroid gland, kidneys, C cells of the thyroid and bone (Leach et al., 2020). Though capable of coupling to multiple heterotrimeric G proteins, most *in vitro* studies of CaR activity, measure G_q/11_-mediated changes in either inositol phosphate metabolism, Ca^2+^_i_ mobilisation or extracellular signal-regulated (ERK) activation, which have helped define CaR pharmacology.

While the CaR has long been known to sense and respond to Ca^2+^_o_, there is now emerging evidence that CaR is sensitive to, and inhibited by, PO_4_ as well (Centeno et al., 2019). Therefore, it is possible that the homeostasis of these two ions may be specifically coordinated. It should be noted that the free, ionised Ca^2+^ concentration in plasma is ∼1.2 mM while the concentration of PO_4_ tends to be a little lower at ∼0.8 mM, though with a wider normal range. Interestingly, this is a similar ratio to that seen for Ca^2+^ and PO_4_ abundance in hydroxyapatite (Ca_5_(PO_4_)_3_OH) and therefore coordinated regulation of their relative plasma concentrations might conceivably be obligatory.

In addition to the proposed inhibitory effect of extracellular PO_4_ ions on CaR activation, in the cytosol, the terminal PO_4_ of ATP can also inhibit CaR signalling, *via* covalent attachment to Ser-875 and Thr-888 residues (i.e., phosphorylation) in the receptor’s intracellular domain (ICD) (McCormick et al., 2010; Binmahfouz et al., 2019). While the dual but distinct roles of extracellular (structural) and intracellular (signalling/metabolic) Ca^2+^ and PO_4_ may be purely an example of energetic efficiency (i.e., multi-tasking with the same, simple elementary chemicals), this nevertheless represents an interesting biological parallel. And more so that for CaR, Ca^2+^_o_ is stimulatory while PO_4_ is inhibitory.

## Orthosteric and allosteric CaR modulators

The CaR exhibits promiscuous pharmacology sensing a broad range of ligands in addition to Ca^2+^ (Leach et al., 2020). Some CaR ligands act as orthosteric agonists, e.g., Mg^2+^ and spermine. Other CaR ligands act as positive allosteric modulators (PAMs; e.g., L-amino acids and calcimimetics) or negative allosteric modulators (NAMs; e.g., calcilytics) (Leach et al., 2020). Structurally, PAMs act by stabilising the CaR’s active conformation, while NAMs stabilise the inactive conformation (Leach et al., 2015). Interestingly, H^+^ and Na^+^ ions, at least at high concentrations are, in effect, NAMs of the CaR. But we have recently shown that PO_4_ ions are also inhibitory for CaR, acting potentially as non-competitive partial CaR antagonists. Here we set out the structural evidence for this (Geng et al., 2016; Zhang et al., 2016) followed by the functional (Centeno et al., 2019; Goodman et al., 2022).

## CaR extracellular domain

**FIGURE 1:**
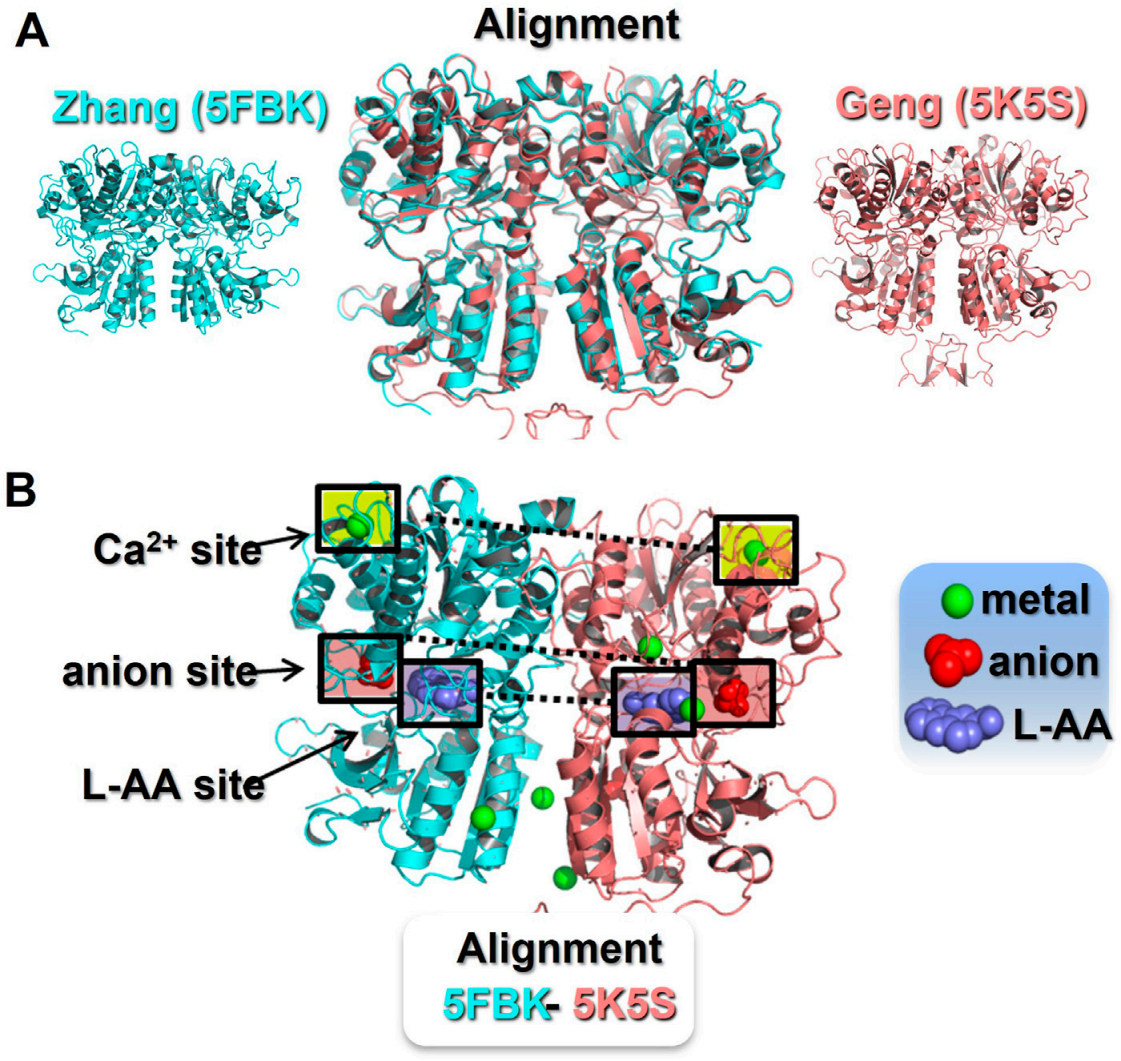
Identification of ionic binding sites in the CaR extracellular domain. *Panel* **(A)** Sequence alignment and structural superposition of the currently available CaR ECD models in the active conformation. The Zhang et al. (2016) model (5FBK) is shown in blue (left) and the Geng et al. (2016) model (5K5S) is shown in pink (right). In the middle, an alignment of both models reveals an almost identical CaR ECD structure with minor differences. Pymol root mean square deviation (RMSD) 0.5, after 5 cycles of iteration. *Panel* **(B)** Active conformation of the CaR ECD with reported ligand binding sites. The Zhang model (5FBH) is shown as the left monomer, with the Geng model (5K5S) on the right. The Zhang model describes three Ca^2+^-binding sites, one in the upper domain and two in the lower domain facing the interface between monomers. At the same locations, the Geng model describes two Ca^2+^-binding sites, but two additional Ca^2+^-binding sites in the cleft between the upper and lower domains. Both models identified a common L-amino acid binding site and a common anion binding site, both located at the cleft between the upper and lower domains. In addition, the Geng model includes an anion binding site located in the lower domain. The ligand binding sites highlighted in boxes are those found in both crystal models. L-AA, L-amino acid.

Two groups have now generated four crystal models of the CaR’s extracellular domain (ECD) (Geng et al., 2016; Zhang et al., 2016) and more recently these structures have been largely confirmed by Cryo-EM (Gao et al., 2021). Three of these crystal models were obtained in the active conformation, while the fourth was obtained in the CaR’s inactive conformation (Geng et al., 2016). Sequence alignment followed by structural superpositioning of the CaR ECD crystal structures (active conformation) confirm the similarity of the two groups’ models, despite differences in their crystallisation environments (Figure 1). That said, the two models still exhibit significant differences in their predicted ligand binding sites and occupancy.

For example, Zhang et al. (2016) reported three metal binding sites (occupied by Mg^2+^ and Gd^3+^), one orthosteric L-aromatic amino acid binding site (occupied by L-1,2,3,4-tetrahydronorharman-3-carboxylic acid (TNCA), an L-Trp derivate), and one potential anion binding site (occupied by bicarbonate). In contrast, the CaR active conformation reported by Geng et al. (2016) suggested four metal binding sites (occupied by Ca^2+^), one orthosteric L-aromatic amino acid binding site (occupied by L-Trp itself), and two anion binding sites (occupied by PO_4_; see Figure 1). In addition, Geng’s inactive model revealed two additional anion binding sites (here occupied by SO_4_) (Geng et al., 2016). These discrepancies could be explained, in part, by the different crystallisation environments. That is, Zhang et al (2016) employed Mg^2+^ and Gd^3+^ in abundance, but with less Ca^2+^ and no L-aromatic amino acids, whereas Geng et al. (2016) included Ca^2+^ and L-Trp in abundance in their crystallisation buffer, and also PO_4_ and SO_4_. Given the greater potency and abundance of Ca^2+^ over Mg^2+^ *in vivo*, the metal binding sites appear more likely to be physiological Ca^2+^-binding sites. Also, based on previous literature, L-Trp and other aromatic amino acids are presumed to be the more likely ligands for the orthosteric L-aromatic amino acid-binding site, rather than TNCA (Mun et al., 2004; Conigrave and Hampson, 2006), though this remains to be confirmed.

## Anion binding sites in CaR

The two crystal models identified four anion binding sites in the CaR’s ECD for the first time. However, it is the anion-binding sites present in the inactive conformation of the Geng et al (2016) model that we believe to be of particular interest. This is because the two, closely-associated anion binding sites appear to play an important role in the stabilisation of the inactive conformation. The first site mainly involves Arg-62 and Tyr-63, while the second involves Arg-66, Arg-69, Thr-412, and Arg-415. When unbound, Arg-62 and Arg-66 may mediate interactions that stabilise the closure of the Venus flytrap (VFT) domain. These interactions, a hydrogen bond (R66-S301) and a salt bridge (R62-E277), are directly breakable by anion binding to the two sites, which would then reduce the free energy needed for the VFT to open and to change to an inactive conformation (Geng et al., 2016).

## Inorganic phosphate homeostasis

Serum PO_4_ levels vary between 0.8 and 1.2 mM in healthy adults, which includes diurnal variation (Kemp et al., 1992). PO_4_ homeostasis is largely determined by the kidney because of PTH- and/or FGF23-mediated phosphaturia (Agoro and White, 2023), though PTH also increases 1α-hydroxylation of 25(OH)-vitamin D thus affecting intestinal PO_4_ absorption (Bergwitz and Juppner, 2010; Komaba and Fukagawa, 2016; Ide et al., 2018). Together, PTH/PTH1R and FGF23/Klotho pathways coordinate to maintain PO_4_ homeostasis (Shimada et al., 2004; Quinn et al., 2013; Kawakami et al., 2017; Fan et al., 2018; Ide et al., 2018). In chronic kidney disease (CKD) however, decreased phosphaturia commonly results in hyperphosphataemia as well as accumulation of calciprotein particles (CPPs), which are associated with soft-tissue calcification, especially vascular calcification and increased risk of death (Block et al., 2004; Tentori et al., 2008; Streja et al., 2014). Crucially, how mammals sense changes in their serum PO_4_ concentration remains unclear (Komaba and Fukagawa, 2016) though the presence of one or more PO_4_ sensors in bone and parathyroid cells, to regulate PTH and FGF23 release, appears likely.

The stimulatory effect of high PO_4_ concentration on PTH secretion has been demonstrated repeatedly *in vivo* and *in vitro* (Almaden et al., 1996; Nielsen et al., 1996; Slatopolsky et al., 1996; Almaden et al., 2003; Rodriguez et al., 2005), but without a clear linking mechanism. Interestingly, when studied *ex vivo*, the PO_4_ effect on PTH secretion was only observed in intact parathyroid tissue preparations but not in dispersed cells, where CaR expression becomes quickly reduced (Nielsen et al., 1996). Patients with secondary hyperparathyroidism (SHPT) show a left-shift in their PTH-Ca^2+^ curve, indicating that higher levels of serum Ca^2+^ are needed to activate CaR-mediated inhibition of PTH secretion (Rodriguez et al., 2005). Our hypothesis for this left-shift in the PTH-Ca^2+^ curve is that the hyperphosphataemia of CKD will promote the direct binding of PO_4_ to the CaR stabilising its inactive conformation and thus permitting increased PTH secretion. Indeed, we have shown that over its pathophysiological concentration range for CKD, PO_4_ inhibits CaR signalling in transfected human embryonic kidney (HEK-293) cells (Centeno et al., 2019). More specifically, PO_4_ lowers the efficacy of Ca^2+^_o_ at the CaR (i.e., E_max_) as opposed to altering the CaR’s sensitivity to Ca^2+^_o_ (EC_50_). As such, the PO_4_ appears to act as a non-competitive CaR (partial) antagonist. Furthermore, raising buffer PO_4_ concentrations rapidly induced PTH secretion from primary human parathyroid cells and from murine parathyroid glands *ex vivo*. The rapid and reversible nature of this PO_4_ effect is indicative of a receptor-mediated event. Mutation of CaR residue Arg-62 (expressed in HEK-293 cells), overcame the inhibitory effect of the added PO_4_ suggesting that the Arg-62 residue may be the PO_4_ binding site, or at least a critical part of it. As mentioned earlier, CaR^R62^ was reported by Geng et al. (2016) to be a PO_4_ binding site present in the inactive conformation of the ECD but not its active conformation. Thus, the current functional data supports this structural prediction so far.

Following publication of the idea that the CaR itself serves as a parathyroid PO_4_ sensor, the clinical trials that had previously demonstrated efficacy for Cinacalcet and Etelcalcetide (used to lower PTH levels in end-stage renal disease) were reanalysed with regards to the prevailing serum PO_4_ concentrations in the patients (Goodman et al., 2022). Subjects were grouped according to whether their serum PO_4_ concentrations were above or below one of three different serum PO_4_ thresholds and these designations were dynamic over time, depending on whether their serum PO_4_ had risen above or dropped below the given threshold in the intervening time. By analysing the calcimimetic responses this way, it was found that calcimimetic-induced decreases in serum PTH levels were attenuated in subjects with higher serum PO_4_ concentrations. The inhibitory effect of high PO_4_ was quite modest for Etelcalcetide though more marked for Cinacalcet, especially at higher PO_4_ concentrations (Goodman et al., 2022). We would argue that, teleologically, it would make sense for PO_4_ to be able to moderate Ca^2+^-induced CaR activity/suppression of PTH secretion but not to be able to ablate it. For example, if one experienced simultaneous hypercalcaemia and hyperphosphataemia then without an inhibitory input from PO_4_, the high Ca^2+^_o_ could maximally suppress PTH secretion abrogating the phosphaturia needed to resolve the hyperphosphataemia. By blunting this Ca^2+^_o_-induced suppression of PTH secretion, the mineral homeostatic system can resolve both issues, albeit potentially less quickly with regards to Ca^2+^. However, if high PO_4_ concentration could suppress CaR activity completely, thus acting effectively as a potent calcilytic, then the additional PTH-induced phosphaturia would resolve the hyperphosphataemia but would worsen the hypercalcaemia, perhaps even dangerously so. Therefore, it might be that as a non-competitive partial antagonist, elevated PO_4_ concentrations could integrate with Ca^2+^ to achieve the optimal PTH secretion for both minerals and not just for Ca^2+^. Furthermore, pathophysiological PO_4_ concentration also partially attenuated the effect of spermine, an endogenous polyamine and CaR agonist (Centeno et al., 2019). Thus, by disrupting the maintenance of VFT closure, PO_4_ may represent a general attenuator of positive CaR modulation, by acting as a non-competitive CaR antagonist.

## Inhibition of CaR by intracellular phosphorylation

Although CaR may couple to a broad range of heterotrimeric G protein families (Leach et al., 2020), it is CaR-induced Gα_q/11_ activation that has been most studied and is likely of the greatest importance for its biological functions. In mice, ablation of Gα_q/11_ results in a phenotype closely resembling that of the CaR knockout (Wettschureck et al., 2007). Similarly, in humans, some gain-of-function Gα_11_ mutations result in autosomal dominant hypocalcaemia (ADH) type-2 while some loss-of-function Gα_11_ mutations produce a familial hypocalciuric hypercalcemia (FHH)-like condition (Nesbit et al., 2013).

Initially, five putative protein kinase C (PKC) phosphorylation sites were identified in CaR, two in the first and third intracellular loops (Thr-646 and Ser-794) and three in the intracellular tail (Thr-888, Ser-895 and Ser-915) (Bai et al., 1998). Mutation of the two intracellular loop residues to non-phosphorylatable residues had no detectable effect on CaR activation, whereas mutation of CaR^T888^, CaR^S895^ and CaR^S915^ increased CaR responsiveness, but with the greatest effect resulting from CaR^T888^ mutation (Bai et al., 1998). Indeed, multiple studies have identified CaR^T888^ as the key PKC phosphorylation site in the CaR (Bai et al., 1998; Davies et al., 2007; Young et al., 2014). Furthermore, the identification of a family with ADH having a novel missense mutation in the PKC phosphorylation site Thr-888 (CaR^T888M^) provides evidence for the physiological importance of CaR^T888^ in humans (Lazarus et al., 2011).

Oscillatory Ca^2+^_i_ signalling is usually ascribed to the IP_3_ receptor (IP_3_R) and how it becomes inhibited by the very rise in Ca^2+^_i_ concentration that it mediates. As the cytosolic Ca^2+^ is returned to the Ca^2+^_i_ stores by sarco/endoplasmic reticulum calcium ATPase (SERCA), the IP_3_R then reopens permitting the next cycle of Ca^2+^_i_ release (Woll and Van Petegem, 2022). However, CaR-induced Ca^2+^_i_ oscillations may also be explained by the existence of cycles of PKC-dependent GPCR phosphorylation and dephosphorylation. This idea was first proposed for metabotropic glutamate receptor 5 (mGluR5)-induced Ca^2+^_i_ oscillations (Nakahara et al., 1997; Bradley et al., 2009). Since CaR shares significant structural and sequence homology with mGluR5, it was then proposed that CaR might also share similarities in terms of Ca^2+^_i_ signalling (Young et al., 2014). In agreement with this, different studies have shown that agonist-induced PKC activation leads to CaR phosphorylation, mostly at CaR^T888^, which uncouples the receptor from its G_q/11_/PLC_β_ signalling and thus inhibits Ca^2+^_i_ release (McCormick et al., 2010; Ward and Riccardi, 2012). In contrast, inhibition of PKC results in decreased levels of CaR^T888^ phosphorylation resulting in CaR reactivation and a subsequent rise in Ca^2+^_i_ mobilisation. Overall, these alternating cycles of CaR^T888^ phosphorylation and dephosphorylation might underlie CaR-induced Ca^2+^_i_ oscillations (Conigrave and Ward, 2013) integrated in some way with the action of the IP_3_R.

**FIGURE 2:**
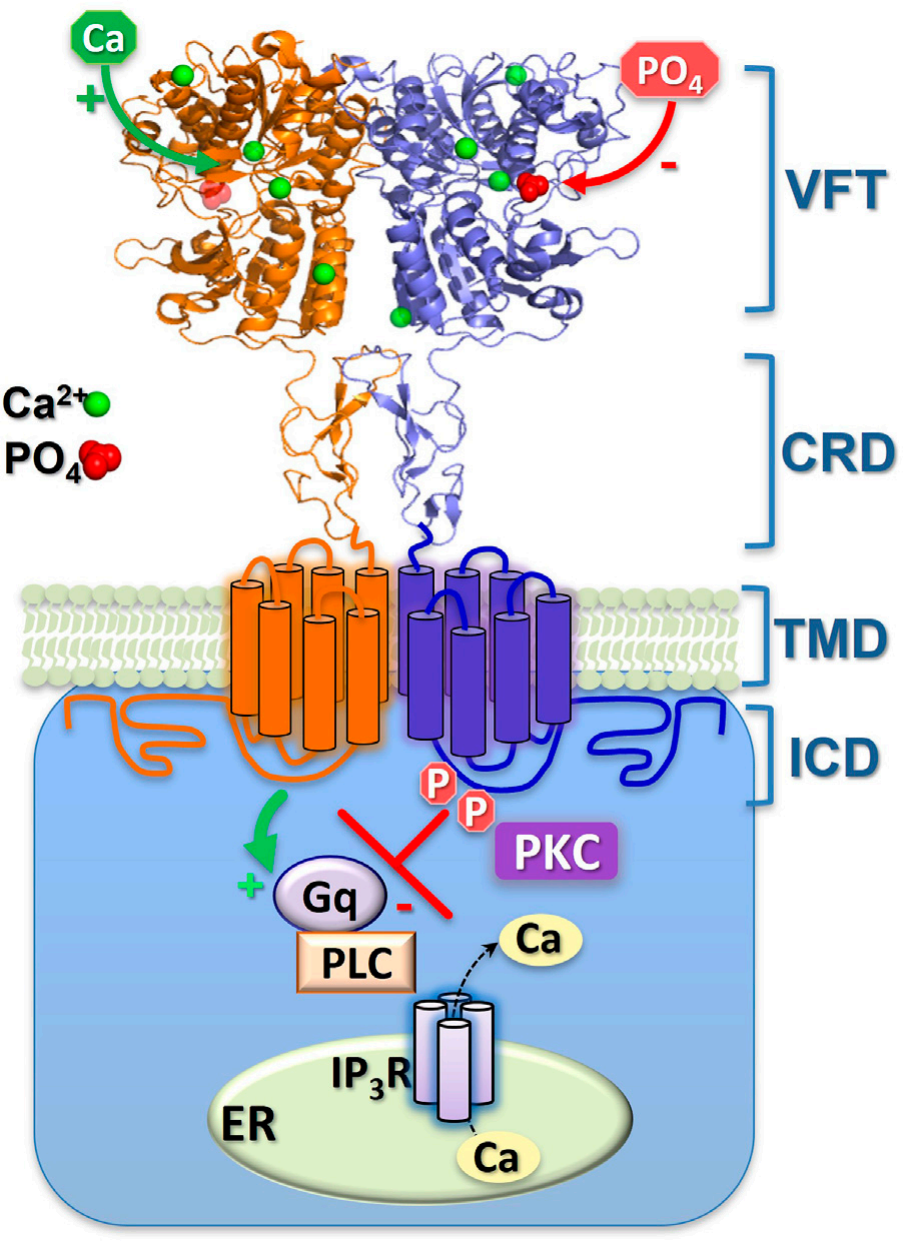
Schematic representation of CaR inhibition by extracellular phosphate ions and by intracellular, covalent phosphorylation. The binding-sites of the (activating) Ca^2+^ and (inhibitory) phosphate (PO_4_) ions shown here are approximate, though their 4:1 ratio is consistent with both the crystal models and functional Hill coefficients. Sustained phosphorylation (P) of ICD residues CaR^S875^ and CaR^T888^ supresses Ca^2+^_i_ mobilisation. Episodic dephosphorylation of CaR^T888^ (at least) permits Ca^2+^_i_ oscillations, while continuous dephosphorylation of these sites elicits enhanced, sustained Ca^2+^_i_ mobilisation. The two monomers of the CaR homodimer are shown either as orange or blue. G_q_, G protein-q/11; PLC, phospholipase C; IP_3_R, IP_3_ receptor; ER, Ca^2+^ stores of the endoplasmic reticulum; PKC, protein kinase C. The CaR domains shown are the venus flytrap (VFT), cysteine-rich domain (CRD), transmembrane domain (TMD) and intracellular domain (ICD).

The residue believed most likely to be responsible for the PKC-mediated inhibition of mGluR5 is Ser-839 (Kim et al., 2005) since mGluR5^S839A^ does not exhibit Ca^2+^_i_ oscillations whereas wild-type mGluR5 does (Kim et al., 2005). Interestingly, mGluR5^S839^ aligns not with CaR^T888^ but with CaR^S875^, which was not initially considered a likely PKC site. However, mutation of CaR^S875^ to alanine increased CaR sensitivity to Ca^2+^, while its mutation to aspartate (a phospho-mimetic site) decreased CaR sensitivity to Ca^2+^, suggesting that CaR^S875^ is another phosphorylation site with an inhibitory action on CaR signalling (Binmahfouz et al., 2019) (Figure 2).

In fact, human CaR has 54 serine and threonine residues in either its intracellular domain (ICD) or intracellular loops (Garrett et al., 1995). Depending on the phosphosite prediction software used, at least 17 of these could be phosphorylation sites, though the total number could be as high as 40. The most likely phosphorylation sites are those serine/threonine residues present in the juxtamembrane region of the ICD, as opposed to those in the carboxyl-terminus. While CaR^T888A^ exhibits enhanced, less oscillatory signalling than for wild-type CaR, cotreatment with a PKC inhibitor can produce a further enhancement of signalling indicating that CaR^T888^ is not the sole target of PKC action (Garrett et al., 1995; Binmahfouz et al., 2019). In contrast, the CaR^S875A/T888A^ double mutation elicits completely non-oscillatory signalling, the same as occurs with PKC inhibition, suggesting that dephosphorylation of both sites is required to abolish the inhibitory effect of PKC (Binmahfouz et al., 2019). Indeed, it has been suggested that the precise pattern of phosphorylation on any given GPCR could vary depending on the cellular context. The so-called “phospho-barcode” hypothesis posits that different phosphorylation “barcode” patterns could elicit distinct downstream signalling outcomes (Tobin et al., 2008; Yang et al., 2017).

## Ca^2+^-sensing by the headless CaR

In this review, we have set out how PO_4_ may inhibit CaR activity both as an extracellular bound anion, but also as an intracellular moiety resulting from phosphorylation. However, there is also recent evidence that raises fascinating questions about the evolutionary purpose of the CaR’s ECD. Synthetic calcimimetics bind within the CaR’s transmembrane domain (TMD), as demonstrated by the observation that their effect is maintained in “headless” CaR mutants lacking the ECD (Hauache et al., 2000; Ray and Northup, 2002; Mun et al., 2004; Rey et al., 2005). Interestingly, Ca^2+^ sensitivity is also retained in these headless mutants, indicating the presence of additional orthosteric Ca^2+^ binding sites in the TMD (Petrel et al., 2004). Indeed, in a headless CaR that also contained the mutation CaR^T888A^ (i.e., where an inhibitory phospho-site was replaced by a non-phosphorylatable alanine), the Ca^2+^_o_ sensitivity was restored to levels not dissimilar to those seen in wild-type CaR (Binmahfouz et al., 2019). This suggests that the CaR ECD is not essential for Ca^2+^_o_ sensitivity *per se*, and thus it might be hypothesised that the ECD a) fine-tunes Ca^2+^_o_-sensitivity to make CaR more physiologically optimal for animal Ca^2+^_o_ homeostasis and/or b) provides for regulation by a range of other physiological modulators (e.g., PO_4_, L-amino acids) making for more sophisticated mineral homeostatic integration. Therefore, further work will be needed to decipher the precise role(s) of the CaR’s ECD.

## Conclusion

The CaR is not merely a Ca^2+^_o_-sensor and Ca^2+^_o_ homeostasis controller but can sense other endogenous ligands, such as phosphate, thus permitting it to integrate mineral homeostasis broadly.
